# Stage-dependent associations between bariatric surgery and chronic kidney disease outcomes in an inpatient database study

**DOI:** 10.1186/s12882-026-05221-4

**Published:** 2026-07-31

**Authors:** Brent Tai, Chijioke Okonkwo, Yaroslav Zuyev, Derek Snyder

**Affiliations:** BayCare Health System, 3231 McMullen Booth Rd, Safety Harbor, Fl 34695 USA

**Keywords:** Chronic kidney disease, Bariatric surgery, End-stage renal disease, Obesity, Type 2 diabetes, CKD progression, Metabolic surgery, National inpatient sample, Propensity score matching, Cardiometabolic risk factors

## Abstract

**Background:**

Bariatric surgery improves obesity-related metabolic dysfunction linked to chronic kidney disease (CKD), including diabetes, hypertension, and systemic inflammation. However, whether its association with CKD differs across disease stages and cardiometabolic risk profiles remains unclear. We examined stage-specific associations between bariatric surgery and CKD in a nationally representative cohort.

**Methods:**

We conducted a cross-sectional analysis using the 2022 Healthcare Cost and Utilization Project Nationwide Inpatient Sample. Adult hospitalizations were categorized by history of bariatric surgery. CKD outcomes included no CKD, stage 3, stages 4–5, and end-stage renal disease (ESRD). Multivariable logistic regression estimated adjusted odds ratios (aORs). Effect modification was assessed using interaction and stratified analyses. Sensitivity analyses included covariate exclusion, inverse probability of treatment weighting (IPTW), and propensity score matching (PSM).

**Results:**

Among 6,578,372 hospitalizations, 66,776 (1.0%) had a history of bariatric surgery. After adjustment, bariatric surgery was associated with lower odds of any CKD (aOR 0.92, 95% CI 0.87–0.98) and stages 4–5 CKD (aOR 0.74, 95% CI 0.63–0.86), but not stage 3 CKD (aOR 1.02, 95% CI 0.95–1.10) or ESRD (aOR 0.92, 95% CI 0.81–1.04). The association with advanced CKD was more pronounced among patients with obesity (aOR 0.70) and diabetes (aOR 0.69), whereas no significant association was observed among patients without these conditions; the association was also stronger in females than in males. Estimates were directionally consistent across sensitivity analyses, although effect sizes varied (IPTW OR 0.87; PSM OR 0.56).

**Conclusions:**

Bariatric surgery was associated with lower odds of advanced CKD, with associations concentrated in metabolically high-risk populations. These findings suggest that the relationship between bariatric surgery and CKD differs by disease stage and may be most evident in more advanced disease, rather than early-stage CKD or ESRD.

**Supplementary Information:**

The online version contains supplementary material available at https://doi.org/10.1186/s12882-026-05221-4.

## Introduction

Chronic kidney disease (CKD) is a major public health problem associated with substantial morbidity, mortality, and healthcare utilization worldwide. The global burden of CKD continues to rise, driven in large part by increasing prevalence of obesity, diabetes, and hypertension—key risk factors that contribute to both the development and worsening severity of kidney disease [1–3]. Importantly, CKD is not a homogeneous condition; more advanced stages are associated with markedly increased risk of cardiovascular events, kidney failure, and death [4–5].

Bariatric surgery has emerged as an effective intervention for severe obesity and related metabolic disorders, leading to sustained weight loss and significant improvements in glycemic control, blood pressure, and systemic inflammation [6–7]. Beyond its metabolic effects, growing evidence suggests that bariatric surgery is associated with improvements in kidney-related outcomes, including reductions in albuminuria and stabilization of kidney function [8–9]. However, the extent to which these associations differ across stages of CKD remains unclear.

Prior studies evaluating the relationship between bariatric surgery and CKD outcomes have yielded mixed results. While some investigations suggest an association with lower risk of adverse kidney outcomes, others have reported limited or inconsistent findings, particularly for advanced outcomes such as end-stage renal disease (ESRD). A key limitation of existing literature is the tendency to treat CKD as a single outcome, without accounting for potential heterogeneity across disease stages [10]. As a result, it remains uncertain whether the association between bariatric surgery and CKD is uniform across the disease spectrum or varies by stage.

Furthermore, the influence of patient-level characteristics, particularly cardiometabolic risk factors such as obesity and diabetes, on the association between bariatric surgery and CKD outcomes has not been fully elucidated. Understanding whether these associations differ across risk profiles is important for identifying patient populations in whom such associations may be most pronounced.

In this study, we used a large, nationally representative inpatient administrative dataset to examine the association between prior bariatric surgery and CKD outcomes. We specifically evaluated stage-specific differences across CKD severity and assessed effect modification by key clinical characteristics. We hypothesized that the association between bariatric surgery and CKD would vary across disease stages, with stronger associations observed for advanced CKD compared with earlier stages. We further hypothesized that these associations would be more pronounced among patients with obesity and diabetes, in whom metabolic and inflammatory pathways targeted by bariatric surgery play a central role in CKD pathophysiology.

## Methods

### Study design, data source, and population

We conducted a retrospective cross-sectional analysis of an inpatient administrative database using the 2022 Healthcare Cost and Utilization Project (HCUP) Nationwide Inpatient Sample (NIS), the largest publicly available all-payer inpatient database in the United States. The NIS approximates a 20% stratified sample of hospital discharges and includes both patient- and hospital-level data [11]. When appropriate discharge weights are applied, the NIS enables generation of nationally representative estimates. As the dataset contains de-identified information, this study was considered exempt from institutional review board oversight by BayCare Health System (Inquiry determination dated October 22, 2025).

We included adult hospitalizations (age ≥ 18 years). Hospitalizations with missing covariates were included in primary regression analyses but excluded from analyses requiring complete-case data, including propensity score methods. Hospitalizations were categorized based on the presence or absence of a history of bariatric surgery using ICD-10-CM code Z98.84. The complete ICD-10-CM codes used in this study are presented in Table S1.

### Exposure, outcomes, and covariates

The primary exposure was prior bariatric surgery. The primary outcome was advanced chronic kidney disease (CKD), defined as CKD stages 4–5. Secondary outcomes included any CKD, CKD stage 3, and end-stage renal disease (ESRD). Cases coded as unspecified CKD (N18.x not otherwise specified) were excluded from stage-specific analyses.

Covariates were selected a priori based on clinical relevance and potential confounding. These included demographic variables (age, sex, race/ethnicity), socioeconomic variables (median household income quartile and primary payer), cardiometabolic comorbidities (diabetes, obesity, and dyslipidemia), cardiovascular comorbidities (heart failure, coronary artery disease, and peripheral vascular disease), additional comorbidities (chronic obstructive pulmonary disease, tobacco use, and liver disease), and hospital characteristics (region, bed size, and teaching status). Hypertension was identified but excluded from multivariable models due to its close clinical overlap with CKD-related and cardiometabolic variables, to avoid overadjustment and redundancy. Multicollinearity assessment using variance inflation factors (VIFs) demonstrated low levels of collinearity among covariates (all VIFs < 2).

### Statistical analysis

Baseline characteristics were summarized using means with standard deviations for continuous variables and frequencies with percentages for categorical variables. Differences between groups were assessed using standardized mean differences (SMDs), with values > 0.1 indicating meaningful imbalance.

Multivariable logistic regression models were used to estimate adjusted odds ratios (aORs) and 95% confidence intervals (CIs) for the association between bariatric surgery and CKD outcomes. Models were adjusted for all covariates described above. Analyses incorporated survey weights to account for the complex sampling design of the NIS, and odds ratios were estimated using a quasibinomial distribution with Wald confidence intervals.

To evaluate heterogeneity across CKD severity, separate logistic regression models were constructed for each CKD category (stage 3, stages 4–5, and ESRD), with hospitalizations without CKD serving as the reference group.

Effect modification was assessed by including interaction terms between bariatric surgery and selected variables (obesity, sex, age, and diabetes), followed by stratified analyses to estimate subgroup-specific associations.

Sensitivity analyses were performed to assess robustness, including sequential exclusion of individual covariates and alternative model specifications.

Propensity score methods were also applied. Inverse probability of treatment weighting (IPTW) was performed using propensity scores estimated from logistic regression including all covariates, with stabilized weights applied and covariate balance assessed using standardized mean differences. Propensity score matching (PSM) was conducted using one-to-one nearest-neighbor matching without replacement, with balance assessed using standardized mean differences. All analyses were performed using R statistical software (version 4.5.3).

## Results

### Study population and baseline characteristics

A total of 6,578,372 hospitalizations were included, of which 66,776 (1.0%) had a history of bariatric surgery (Table 1). Patients with prior bariatric surgery were older (mean age 56.4 vs. 49.9 years; standardized mean difference [SMD] 0.29), indicating meaningful baseline imbalance, and were more likely to be female (77.9% vs. 55.1%; SMD 0.50). The bariatric group also had a higher prevalence of obesity (44.5% vs. 15.8%; SMD 0.66), hypertension (40.5% vs. 26.0%; SMD 0.31), diabetes (29.6% vs. 24.3%; SMD 0.12), and liver disease (10.5% vs. 6.1%; SMD 0.16).

**Table 1:** Baseline characteristics of hospitalizations stratified by bariatric surgery status

Variable	Non-Bariatric (*n* = 6,511,596)	Bariatric (*n* = 66,776)	SMD
**Age**,** mean (SD)**	49.94 (27.78)	56.37 (13.80)	0.293
**Female**,** %**	55.1	77.9	0.496
**Race/Ethnicity**,** %**			0.234
White	62.7	69.2	
Black	15.5	15.8	
Hispanic	14.2	11.4	
Asian	3.3	0.6	
Native American	0.7	0.5	
Other	3.6	2.5	
**Median Household Income Quartile**,** %**			0.073
Q1 (lowest)	29.5	27.1	
Q2	25.7	27.1	
Q3	24.2	26.4	
Q4 (highest)	20.6	19.4	
**Primary payer**,** %**			0.239
Medicare	40.2	43.6	
Medicaid	23.7	15.9	
Private	28.7	35.3	
Self-pay	3.9	2.4	
No charge	0.2	0.2	
Other	3.2	2.7	
**Hospital region**,** %**			0.098
Northeast	17.7	20.3	
Midwest	21.4	23.4	
South	40.7	38.5	
West	20.2	17.8	
**Bed size**,** %**			0.035
Small	22.6	24.0	
Medium	28.5	28.2	
Large	49.0	47.8	
**Location/Teaching status**,** %**			0.065
Rural	8.2	6.5	
Urban non-teaching	15.5	15.7	
Urban teaching	76.3	77.7	
**Comorbidities**,** %**			
Diabetes Mellitus	24.3	29.6	0.120
Hypertension	26.0	40.5	0.312
Obesity	15.8	44.5	0.658
Heart Failure	16.9	14.9	0.055
Peripheral Vascular Disease	3.6	2.6	0.059
Coronary Artery Disease	17.4	15.6	0.047
Chronic Obstructive Pulmonary Disease	10.9	9.8	0.036
Dyslipidemia	31.9	34.6	0.058
Smoking	14.2	13.4	0.024
Liver disease	6.1	10.5	0.161

Baseline characteristics of hospitalizations stratified by history of bariatric surgery, with standardized mean differences used to assess between-group balance. Continuous variables are presented as mean (standard deviation) and categorical variables as number (percentage). Standardized mean differences (SMDs) were used to assess differences between groups, with values > 0.1 indicating meaningful imbalance

Racial and socioeconomic differences were observed, with a higher proportion of White patients and greater representation of privately insured individuals in the bariatric group. Differences in hospital characteristics were modest (all SMDs < 0.1).

### Association between bariatric surgery and CKD outcomes

In unadjusted analyses, bariatric surgery was associated with lower odds of any CKD (odds ratio [OR] 0.86, 95% confidence interval [CI] 0.84–0.88), CKD stage 3 (OR 0.88, 95% CI 0.85–0.91), CKD stage 4–5 (OR 0.71, 95% CI 0.67–0.76), and ESRD (OR 0.84, 95% CI 0.80–0.88) (Table 2).

**Table 2:** Association between prior bariatric surgery and chronic kidney disease outcomes

Outcome	*N* (unweighted)	% (unweighted)	*N* (weighted)	% (weighted)	Crude OR (95% CI)	*p*-value	Adjusted OR (95% CI)	*p*-value
No CKD (reference)	4,761,085	85.5%	23,805,417	85.5%	—	—	—	—
CKD stage 3	473,856	8.5%	2,369,278	8.5%	0.88 (0.85–0.91)	< 0.001	1.02 (0.95–1.10)	0.587
CKD stages 4–5	131,644	2.4%	658,220	2.4%	0.71 (0.67–0.76)	< 0.001	0.74 (0.63–0.86)	< 0.001
ESRD	204,735	3.7%	1,023,675	3.7%	0.84 (0.80–0.88)	< 0.001	0.92 (0.81–1.04)	0.186

Unadjusted and multivariable-adjusted odds ratios (ORs) for the association between prior bariatric surgery and chronic kidney disease (CKD) outcomes. Stage-specific analyses compare each CKD category with hospitalizations without CKD as the reference group

**Fig. 1:**
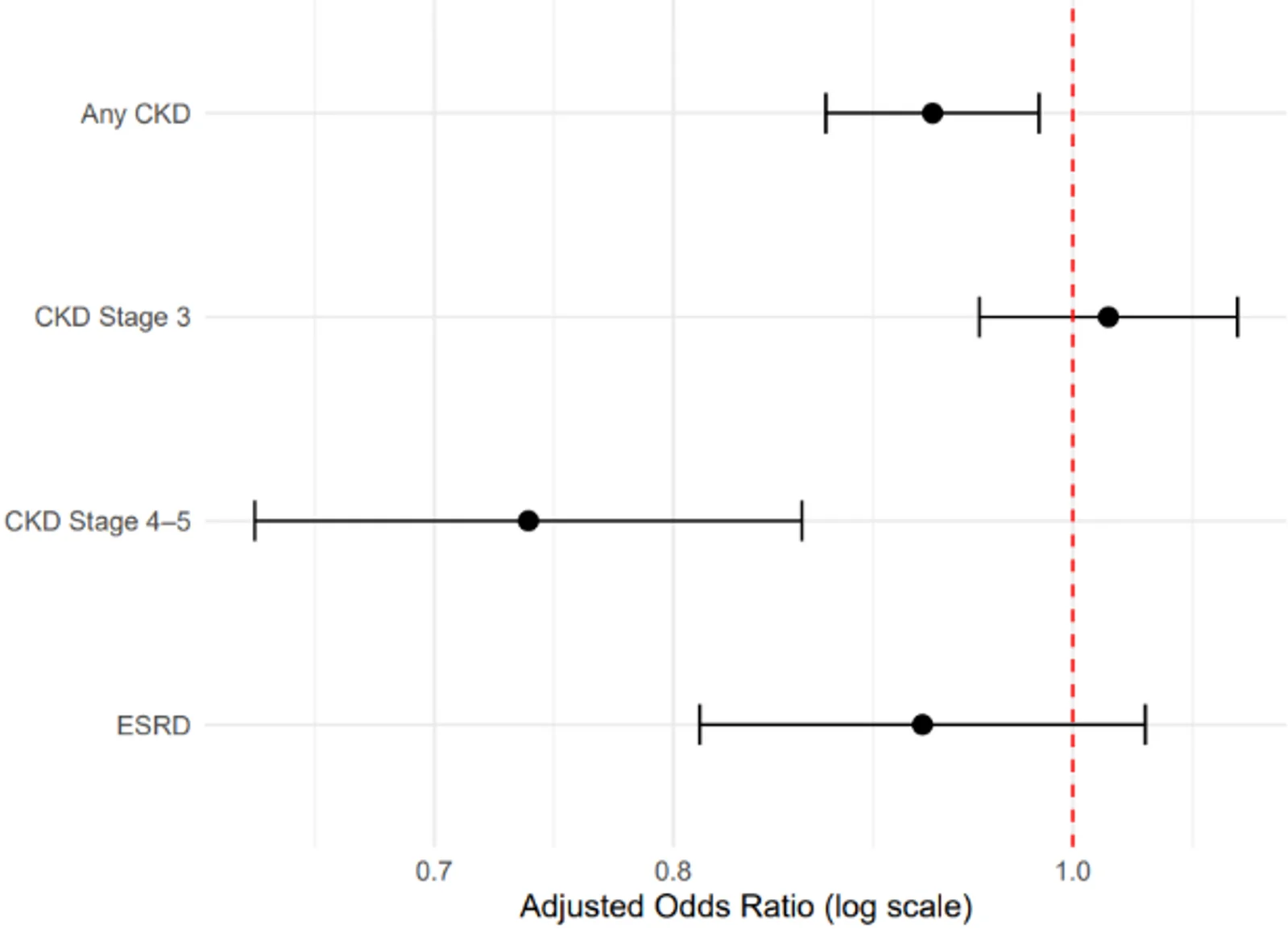
Association between prior bariatric surgery and chronic kidney disease outcomes. Forest plot showing the associations between prior bariatric surgery and chronic kidney disease (CKD) outcomes, including any CKD, CKD stage 3, CKD stages 4–5, and end-stage renal disease (ESRD). The vertical dashed line represents the null value (OR = 1)

After multivariable adjustment, the association with CKD remained statistically significant but attenuated (adjusted OR [aOR] 0.92, 95% CI 0.87–0.98; *p* = 0.010). In contrast, the association with CKD stage 3 was no longer significant (aOR 1.02, 95% CI 0.95–1.10; *p* = 0.587). Bariatric surgery remained associated with significantly lower odds of CKD stage 4–5 (aOR 0.74, 95% CI 0.63–0.86; *p* < 0.001), whereas no significant association was observed for ESRD (aOR 0.92, 95% CI 0.81–1.04; *p* = 0.186). These findings are illustrated in Fig. 1.

A subset of hospitalizations with CKD were classified as unspecified CKD based on ICD-10-CM coding and were excluded from stage-specific analyses.

### Stratified and interaction analyses

**Fig. 2:**
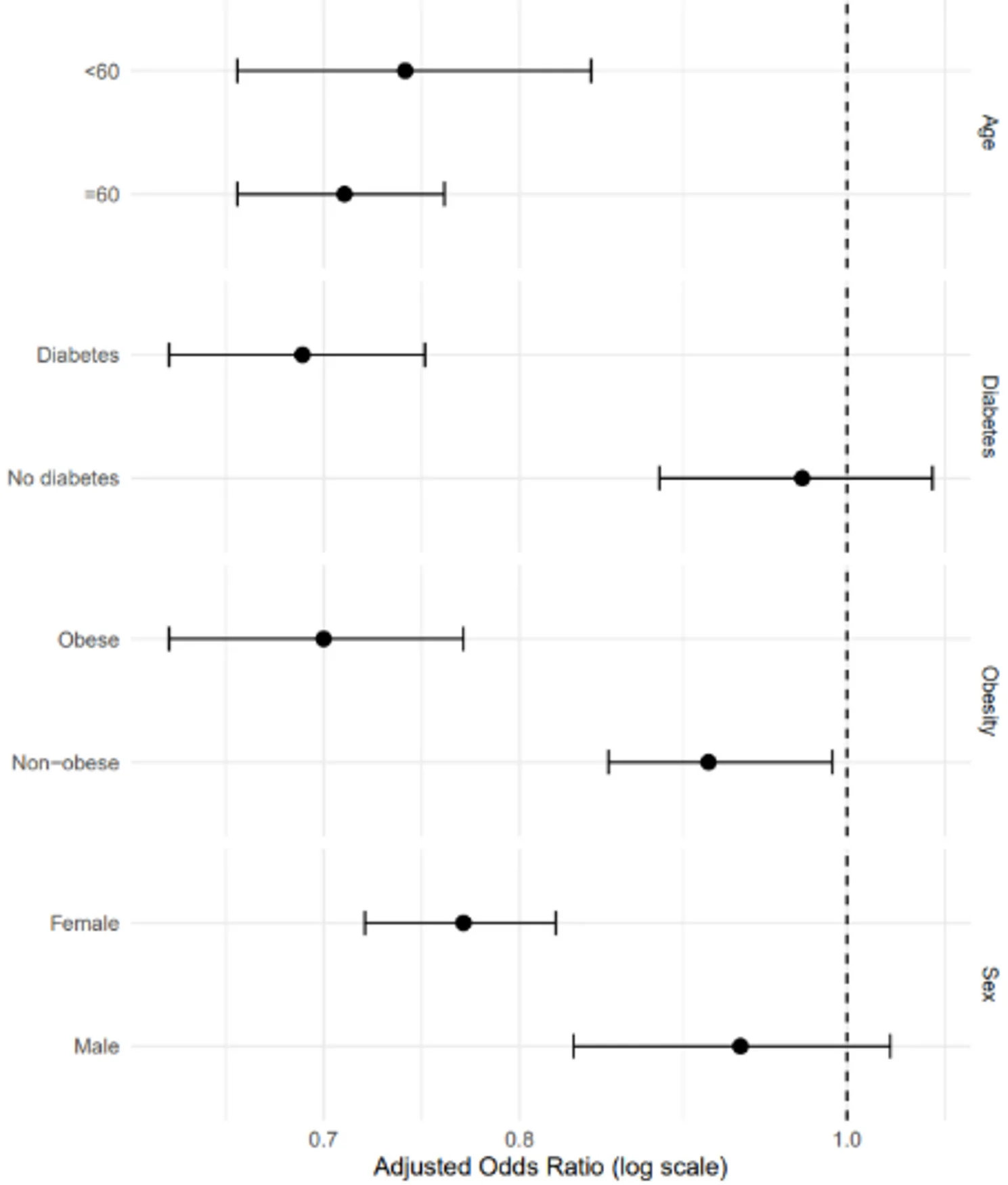
Stratified associations between prior bariatric surgery and advanced chronic kidney disease. Forest plot demonstrating effect modification for the association between prior bariatric surgery and advanced chronic kidney disease across clinically relevant subgroups, including age, diabetes status, obesity, and sex. The vertical dashed line represents the null value (OR = 1)

In stratified analyses, the association between bariatric surgery and CKD stages 4–5 varied across subgroups (Table S2). The association was more pronounced among patients with obesity (aOR 0.70, 95% CI 0.63–0.77; *p* < 0.001) and diabetes (aOR 0.69, 95% CI 0.63–0.75; *p* < 0.001), whereas no significant association was observed among patients without diabetes (aOR 0.97, 95% CI 0.88–1.06; *p* = 0.467). A significant association was also observed among females (aOR 0.77, 95% CI 0.72–0.82; *p* < 0.001), but not among males (aOR 0.93, 95% CI 0.83–1.03; *p* = 0.160). The association was similar across age groups. These findings are visualized in Fig. 2.

Interaction analyses confirmed effect modification by obesity, sex, and age, while no significant interaction was observed with diabetes. Secondary interaction analyses by insurance status demonstrated a stronger protective association among Medicaid patients, with no significant interaction observed for other payer groups (Table S3).

### Sensitivity analyses

Sensitivity analyses demonstrated that the association between bariatric surgery and CKD stage 4–5 was robust to exclusion of individual covariates (Table S4). Adjusted odds ratios ranged from 0.69 to 0.83 across models, indicating that the observed association was not driven by any single confounder.

Additional sensitivity analyses using alternative analytic approaches yielded varying estimates (Table S5). The multivariable-adjusted model showed a significant association (aOR 0.74, 95% CI 0.63–0.86), whereas IPTW analyses attenuated the association (OR 0.87, 95% CI 0.75–1.02). In contrast, propensity score matching demonstrated a stronger association (OR 0.56, 95% CI 0.45–0.69), though based on a smaller matched cohort. Covariate balance before and after IPTW and propensity score matching is shown in Figures S1 and S2, respectively.

## Discussion

In this large, nationally representative cross-sectional study of over 6.5 million hospitalizations, prior bariatric surgery was associated with lower odds of chronic kidney disease (CKD) overall, with important differences by disease stage. After adjustment, no association was observed with CKD stage 3 or end-stage renal disease (ESRD), whereas bariatric surgery remained associated with lower odds of advanced CKD (stages 4–5). These findings highlight a stage-dependent pattern, suggesting that the association between bariatric surgery and CKD varies across the spectrum of disease severity.

Our findings are consistent with prior studies demonstrating that bariatric surgery is associated with improved kidney-related outcomes [9, 12, 13]. Observational and longitudinal studies have reported improvements in kidney function following bariatric surgery [14–16]. Our results extend this literature by demonstrating that these associations are not uniform across CKD stages. Specifically, bariatric surgery was associated with lower odds of advanced CKD but not earlier-stage CKD or ESRD, suggesting that the relationship between bariatric surgery and CKD may differ by disease severity rather than reflecting a uniform association across all stages. This observation may help explain previously inconsistent findings in the literature, which often treat CKD as a homogeneous condition despite substantial heterogeneity across disease stages [10].

The stage-dependent pattern observed in our study is biologically plausible and reflects the central role of metabolic and inflammatory pathways in CKD. Insulin resistance, a key driver of obesity-related metabolic dysfunction, disrupts multiple intracellular signaling pathways, including PI3K/Akt and MAPK cascades, leading to impaired glucose utilization, endothelial dysfunction, and pro-inflammatory gene activation [17]. These processes promote oxidative stress, cytokine-mediated injury (e.g., TNF-α, IL-6), and microvascular damage, all of which contribute to progressive renal fibrosis and nephron loss.

Obesity further amplifies these effects through adipose tissue–driven inflammation, lipotoxicity, and activation of the renin–angiotensin–aldosterone system, resulting in glomerular hyperfiltration and intraglomerular hypertension [18–19]. Bariatric surgery favorably modifies many of these mechanisms by improving insulin sensitivity, reducing systemic inflammation, and restoring metabolic homeostasis [17, 20, 21].

Importantly, CKD represents a heterogeneous condition with distinct phenotypic and metabolic profiles, as demonstrated in recent data-driven studies of diabetic kidney disease [22]. Early-stage CKD (stage 3) encompasses diverse etiologies and variable clinical trajectories, which may dilute detectable associations. In contrast, advanced CKD (stages 4–5) is characterized by more uniform and active disease processes driven by sustained metabolic and inflammatory injury. This framework may explain why bariatric surgery was associated with lower odds of advanced CKD but not earlier stages.

The lack of association with ESRD likely reflects a combination of irreversible structural kidney damage, including extensive nephron loss and fibrosis [18, 23], as well as selection and survival bias within this highly selected population. In addition, ESRD represents a complex clinical endpoint influenced by multiple factors beyond metabolic status, including access to care, referral patterns, and competing risks.

Our stratified analyses further demonstrated that the association between bariatric surgery and advanced CKD was concentrated among patients with obesity and diabetes. This finding is biologically plausible, as these conditions are major drivers of insulin resistance, systemic inflammation, and progressive kidney injury. Bariatric surgery substantially improves glycemic control and metabolic health [20–21], suggesting that its association with CKD may be most pronounced among patients with the highest cardiometabolic burden.

We also observed that the association between bariatric surgery and advanced CKD differed by sex, with a statistically significant association among females but not among males. This may reflect differences in patient selection, baseline characteristics, or postoperative outcomes. Women comprise the majority of bariatric surgery recipients, and sex differences in referral patterns and utilization have been well described [24–25, 26]. Men who undergo bariatric surgery tend to be older and have a greater burden of comorbid disease, which may attenuate observed associations. Prior studies have also reported sex-specific differences in weight-loss and metabolic outcomes following bariatric procedures [27]. These findings should be interpreted cautiously and considered hypothesis-generating, and further studies are needed to evaluate potential sex-specific associations.

This study has several strengths. The use of a large, nationally representative dataset enhances generalizability. Multiple analytic approaches were used to assess robustness; the multivariable-adjusted and IPTW analyses likely provide the most generalizable estimates, whereas propensity score matching, based on a smaller matched cohort, should be interpreted as exploratory. Variation across analytic approaches is consistent with the underlying biological and methodological complexity of cardiometabolic disease, in which multiple interacting pathways may influence observed associations [28, 29]. In addition, evaluation of stage-specific CKD outcomes and effect modification allows for more clinically meaningful interpretation.

Several limitations should be considered. First, this was a cross-sectional analysis of administrative data, and therefore the temporal relationship between bariatric surgery and CKD stage cannot be determined. Second, reliance on ICD-10 coding may result in misclassification, particularly for early-stage CKD, which is often undercoded and may attenuate associations for stage 3 disease. Third, important clinical variables, including body mass index, laboratory measures (e.g., eGFR and albuminuria), duration of diabetes, and use of renoprotective medications, are not available in the NIS database, introducing potential residual confounding. Cardiometabolic outcomes are influenced by complex interactions between traditional and emerging risk factors, including inflammatory and metabolic pathways, many of which are not captured in administrative datasets [30]. Finally, propensity-based analyses were restricted to complete cases, which may introduce selection bias, although consistent findings across analytic approaches support the robustness of results.

## Conclusions

In this large national study, bariatric surgery was associated with lower odds of advanced CKD, but not CKD stage 3 or ESRD. The association was most pronounced among individuals with obesity and diabetes and differed by sex and age, indicating heterogeneity across patient populations. These findings suggest that the relationship between bariatric surgery and CKD is stage-dependent, with potential benefits concentrated in later stages of disease and among metabolically high-risk groups. Our results highlight the importance of considering both disease stage and metabolic risk when evaluating the renal implications of bariatric surgery. Further longitudinal studies are needed to clarify causal pathways and long-term renal outcomes.

## Supplementary Information

Below is the link to the electronic supplementary material.

Supplementary Material 1

Supplementary Material 2

Supplementary Material 3

Supplementary Material 4

Supplementary Material 5

Supplementary Material 6

Supplementary Material 7

## Data Availability

The data that support the findings of this study are available from the Agency for Healthcare Research and Quality, Department of Health and Human Services of the United States. However, restrictions apply to the availability of these data, which were used under license for the current study, and so are not publicly available. Data are, however, available from the author upon reasonable request and with permission of the Agency for Healthcare Research and Quality.
