# Supplementary material for: Stage-dependent associations between bariatric surgery and chronic kidney disease outcomes in an inpatient database study
Source: BMC Nephrol. 2026 Jul 31;27:638. doi: 10.1186/s12882-026-05221-4 (PMC13721188; doi:10.1186/s12882-026-05221-4)
Supplement: Supplementary file 1 [file 12882_2026_5221_MOESM1_ESM.docx]

Figure S1. Covariate Balance Before and After Inverse Probability of Treatment Weighting

Figure S1. Love plot demonstrates standardized mean differences (SMDs) for baseline covariates before and after inverse probability of treatment weighting (IPTW). Red points represent unadjusted differences, and blue points represent IPTW-adjusted differences. The dashed vertical line indicates an SMD threshold of 0.1, with values below this threshold indicating adequate covariate balance.
