# Supplementary material for: Stage-dependent associations between bariatric surgery and chronic kidney disease outcomes in an inpatient database study
Source: BMC Nephrol. 2026 Jul 31;27:638. doi: 10.1186/s12882-026-05221-4 (PMC13721188; doi:10.1186/s12882-026-05221-4)
Supplement: Supplementary file 2 [file 12882_2026_5221_MOESM2_ESM.docx]

Figure S2. Covariate Balance Before and After Propensity Score Matching

Figure S2. Love plot demonstrating standardized mean differences (SMDs) for baseline covariates before and after propensity score matching (PSM). Red points represent unmatched (pre-matching) differences, and blue points represent matched (post-matching) differences. The dashed vertical line indicates an SMD threshold of 0.1, with values below this threshold indicating adequate covariate balance.
