# Supplementary material for: Stage-dependent associations between bariatric surgery and chronic kidney disease outcomes in an inpatient database study
Source: BMC Nephrol. 2026 Jul 31;27:638. doi: 10.1186/s12882-026-05221-4 (PMC13721188; doi:10.1186/s12882-026-05221-4)
Supplement: Supplementary file 3 [file 12882_2026_5221_MOESM3_ESM.docx]

Table S1. ICD-10-CM codes used to define study variables and comorbid conditions

| Variable | ICD-10-CM Codes | Description |
| --- | --- | --- |
| Bariatric surgery (history) | Z98.84 | History of bariatric surgery |
| Chronic kidney disease (CKD) | N18.x | CKD, all stages |
| CKD stage 3 | N18.3x | Moderate CKD |
| CKD stage 4 | N18.4 | Severe CKD |
| CKD stage 5 | N18.5 | Kidney failure |
| End-stage renal disease (ESRD) | N18.6 | ESRD |
| Diabetes mellitus | E10.x, E11.x | Type 1 and type 2 diabetes |
| Obesity | E66.x | Obesity |
| Dyslipidemia | E78.x | Disorders of lipoprotein metabolism |
| Heart failure | I50.x | Heart failure |
| Coronary artery disease (CAD) | I25.x | Chronic ischemic heart disease |
| Peripheral vascular disease (PVD) | I70.x, I73.x | Atherosclerosis and peripheral vascular disease |
| Chronic obstructive pulmonary disease (COPD) | J44.x | COPD |
| Smoking / tobacco use | F17.x, Z72.0 | Nicotine dependence and tobacco use |
| Liver disease | K70–K77 | Diseases of the liver |
| Hypertension (not included in main model) | I10.x | Essential hypertension |

Table S1 presents ICD-10-CM codes in any diagnosis position. Prefix “.x” indicates inclusion of all subcodes. CKD stages were categorized based on ICD-10-CM definitions.
