# Supplementary material for: Stage-dependent associations between bariatric surgery and chronic kidney disease outcomes in an inpatient database study
Source: BMC Nephrol. 2026 Jul 31;27:638. doi: 10.1186/s12882-026-05221-4 (PMC13721188; doi:10.1186/s12882-026-05221-4)
Supplement: Supplementary file 4 [file 12882_2026_5221_MOESM4_ESM.docx]

Table S2. Stratified analyses of the association between prior bariatric surgery and advanced chronic kidney disease

| Subgroup | Adjusted OR (95% CI) | p-value |
| --- | --- | --- |
| Obesity |  |  |
| Obese | 0.70 (0.63–0.77) | <0.001 |
| Non-obese | 0.91 (0.85–0.99) | 0.021 |
| Sex |  |  |
| Female | 0.77 (0.72–0.82) | <0.001 |
| Male | 0.93 (0.83–1.03) | 0.160 |
| Age |  |  |
| <60 | 0.74 (0.66–0.84) | <0.001 |
| ≥60 | 0.71 (0.66–0.76) | <0.001 |
| Diabetes |  |  |
| Diabetes | 0.69 (0.63–0.75) | <0.001 |
| No diabetes | 0.97 (0.88–1.06) | 0.467 |

Table S2. Interaction analyses were performed to assess effect modification in the association between bariatric surgery and advanced chronic kidney disease. Interaction terms between bariatric surgery and selected variables (obesity, sex, age, and diabetes) were included in multivariable logistic regression models. Odds ratios for interaction terms represent the relative difference in the association across levels of each variable.
