# Supplementary material for: Stage-dependent associations between bariatric surgery and chronic kidney disease outcomes in an inpatient database study
Source: BMC Nephrol. 2026 Jul 31;27:638. doi: 10.1186/s12882-026-05221-4 (PMC13721188; doi:10.1186/s12882-026-05221-4)
Supplement: Supplementary file 5 [file 12882_2026_5221_MOESM5_ESM.docx]

Table S3. Secondary interaction analyses by insurance status

| Insurance | Interaction OR (95% CI) |
| --- | --- |
| Medicaid | 0.71 (0.57–0.90) |
| Private | 1.04 (0.90–1.19) |
| Self-pay | 0.79 (0.46–1.38) |
| Other | 1.11 (0.73–1.68) |

Table S3. Secondary interaction analyses evaluating effect modification by insurance status in the association between prior bariatric surgery and advanced chronic kidney disease. Interaction terms between bariatric surgery and insurance status (primary payer) were included in multivariable logistic regression models adjusted for demographic, socioeconomic, clinical, and hospital characteristics. Odds ratios represent the relative difference in the association between bariatric surgery and advanced CKD across insurance categories, with Medicare as the reference group. Estimates for the “No charge” category were not reported due to sparse data and unstable model estimates.
