# Supplementary material for: Stage-dependent associations between bariatric surgery and chronic kidney disease outcomes in an inpatient database study
Source: BMC Nephrol. 2026 Jul 31;27:638. doi: 10.1186/s12882-026-05221-4 (PMC13721188; doi:10.1186/s12882-026-05221-4)
Supplement: Supplementary file 6 [file 12882_2026_5221_MOESM6_ESM.docx]

Table S4. Sensitivity analyses excluding individual covariates for the association between prior bariatric surgery and advanced chronic kidney disease.

| Model | Adjusted OR (95% CI) |
| --- | --- |
| Main model | 0.74 (0.63–0.86) |
| Excluding diabetes | 0.81 (0.77–0.86) |
| Excluding obesity | 0.83 (0.78–0.87) |
| Excluding insurance | 0.81 (0.76–0.86) |
| Excluding age | 0.69 (0.65–0.74) |
| Excluding sex | 0.79 (0.75–0.84) |
| Excluding race | 0.79 (0.75–0.84) |
| Excluding income | 0.81 (0.76–0.86) |
| Excluding dyslipidemia | 0.81 (0.76–0.86) |
| Excluding heart failure | 0.76 (0.72–0.81) |
| Excluding coronary artery disease | 0.81 (0.77–0.86) |
| Excluding peripheral vascular disease | 0.81 (0.76–0.86) |
| Excluding COPD | 0.81 (0.77–0.86) |
| Excluding smoking | 0.82 (0.77–0.87) |
| Excluding liver disease | 0.81 (0.77–0.86) |

Table S4. Each model excluded one covariate from the fully adjusted model to assess the influence of individual variables on the association between bariatric surgery and advanced CKD. All models were adjusted for remaining covariates, including demographic, socioeconomic, clinical, and hospital characteristics. Odds ratios and 95% confidence intervals were estimated using weighted logistic regression with quasibinomial distribution and Wald methods.
