# Supplementary material for: Stage-dependent associations between bariatric surgery and chronic kidney disease outcomes in an inpatient database study
Source: BMC Nephrol. 2026 Jul 31;27:638. doi: 10.1186/s12882-026-05221-4 (PMC13721188; doi:10.1186/s12882-026-05221-4)
Supplement: Supplementary file 7 [file 12882_2026_5221_MOESM7_ESM.docx]

Table S5. Sensitivity Analyses for the Association Between Bariatric Surgery and Advanced Chronic Kidney Disease

| Model | OR (95% CI) | n |  |
| --- | --- | --- | --- |
| Multivariable adjusted | 0.74 (0.63–0.86) | 1,000,000 |  |
| IPTW-weighted | 0.87 (0.75–1.02) | 950,002 |  |
| Propensity score–matched | 0.56 (0.45–0.69) | 19,864 |  |

Table S5. Sensitivity analyses were performed using inverse probability of treatment weighting (IPTW) and propensity score matching (PSM) to evaluate robustness of the association between bariatric surgery and advanced chronic kidney disease. IPTW weights were derived from a propensity score model including demographic, socioeconomic, comorbidity, and hospital-level variables. Stabilized weights were applied, and weighted logistic regression models were used to estimate odds ratios. Propensity score matching was performed using 1:1 nearest-neighbor matching without replacement. Covariate balance was assessed using standardized mean differences. Differences in sample size reflect exclusion of observations with missing data and restriction to matched pairs in the propensity score–matched analysis.
